# Three enzymes governed the rise of O_2_ on Earth

**DOI:** 10.1016/j.bbabio.2024.149495

**Published:** 2024-07-14

**Authors:** Natalia Mrnjavac, Mauro Degli Esposti, Itzhak Mizrahi, William F. Martin, John F. Allen

**Affiliations:** a Department of Biology, Institute for Molecular Evolution, Heinrich Heine University of Duesseldorf, Duesseldorf, Germany; b Center for Genomic Sciences, UNAM Campus de Cuernavaca, Morelos, Mexico; c Department of Life Sciences, Ben-Gurion University of the Negev and the National Institute for Biotechnology in the Negev, Marcus Family Campus, Be’er-Sheva, Israel; d Research Department of Genetics, Evolution and Environment, University College London, Gower Street, London, UK

**Keywords:** Oxygen, Enzymes, Evolution, Great oxidation event, GOE, Terrestrialization, Nitrogenase

## Abstract

Current views of O_2_ accumulation in Earth history depict three phases: The onset of O_2_ production by ~2.4 billion years ago; 2 billion years of stasis at ~1 % of modern atmospheric levels; and a rising phase, starting about 500 million years ago, in which oxygen eventually reached modern values. Purely geochemical mechanisms have been proposed to account for this tripartite time course of Earth oxygenation. In particular the second phase, the long period of stasis between the advent of O_2_ and the late rise to modern levels, has posed a puzzle. Proposed solutions involve Earth processes (geochemical, ecosystem, day length). Here we suggest that Earth oxygenation was not determined by geochemical processes. Rather it resulted from emergent biological innovations associated with photosynthesis and the activity of only three enzymes: 1) The oxygen evolving complex of cyanobacteria that makes O_2_; 2) Nitrogenase, with its inhibition by O_2_ causing two billion years of oxygen level stasis; 3) Cellulose synthase of land plants, which caused mass deposition and burial of carbon, thus removing an oxygen sink and therefore increasing atmospheric O_2_. These three enzymes are endogenously produced by, and contained within, cells that have the capacity for exponential growth. The catalytic properties of these three enzymes paved the path of Earth’s atmospheric oxygenation, requiring no help from Earth other than the provision of water, CO_2_, salts, colonizable habitats, and sunlight.

## 1 Introduction

Molecular oxygen, O_2_, is a stable diradical and a strong oxidant with a midpoint potential of +815 mV for the O_2_/H_2_O couple at pH 7. O_2_ accepts only single electrons in all biological reactions, generating a highly reactive radical (an unpaired electron) both in O_2_ itself and in its interacting substrate during the enzymatic mechanism. O_2_ is so energy-rich as a reaction partner that it undergoes exothermic redox reactions with every element except gold [1]. It has been estimated that 80 % of mineral species known on Earth today owe their origin to biological processes of some type [2]. These processes almost always involve O_2_. Oxygen’s tendency to accept single electrons may also underly most of its geochemical reactions. Oxygen on Earth is produced as a byproduct of water oxidation at the oxygen evolving complex (OEC) of photosystem II in cyanobacteria and plastids. Before cyanobacteria, Earth was devoid of molecular oxygen [3–5]. Production of O_2_ by cyanobacteria changed the world.

By most accounts, O_2_ began to accumulate in Earth’s atmosphere at the Great Oxidation Event, GOE [6–8], roughly 2.4 billion years ago (Ga). There are reports for small amounts (“whiffs”) of O_2_ in slightly older sediments [9], ca. 50 MY (million years) prior to the GOE, based on the redox state of redox-sensitive minerals. The evidence for pre-GOE whiffs [10] has recently been challenged on the basis that O_2_ might have diffused into the redox-sensitive sediment after the GOE [11,12]. Geochemists understandably view the exact timing of events in Earth history as crucial. From the perspective of day-to-day life, 50 MY is admittedly a huge amount of time. But for biologists, the difference between O_2_ being 2.4 Ga or 2.45 Ga old is slight (±2 %), while geologically irrelevant timescales may be crucial. For example, 2 milliseconds is the duration of a Kok cycle [13] of manganese and water oxidation for O_2_ synthesis at the OEC [14]. Although the Kok cycle is of short duration, it has taken place continuously during 2.4 billion years of O_2_ synthesis, once for every O_2_ molecule ever made. There are currently about 10^44^ O_2_ molecules resident in the atmosphere, all made by the OEC, and the time required for the OEC to make O_2_ from H_2_O is shorter by a factor ~ 10^20^ than the duration of its presence on the planet. This difference in scale highlights a property of biological mechanisms. Enzymes are present in large numbers and catalyze vast numbers of individual reactions, one molecule at a time. This contrasts with the lower frequency and global impact of individual geological events such as volcanism or subduction.

Direct evidence for the history of O_2_ accumulation on Earth is derived from the geochemical record. Biologists can use geochemical benchmarks to make indirect inferences about the history of O_2_ based on phylogenetic trees and molecular clocks, but the only direct evidence for O_2_ accumulation is in rocks. Today, geochemists distinguish three phases in O_2_ history. The first phase was the onset of O_2_-production by cyanobacteria prior to the Great Oxidation Event (GOE) 2.4 billion years (Ga) ago [6]. The GOE conventionally marks the time when O_2_ became a stable component of the atmosphere, rising above trace levels or 0.001 % present atmospheric levels (PAL) according to the disappearance of non-mass-dependent sulfur isotope fractionation in the sedimentary record [3,15]. The second phase in O_2_ history was one of almost 2 billion years of stasis: following the GOE, oxygen did not rise to modern levels, but remained at about ~1 % of present atmospheric levels (PAL) [3,4,7,8] until the onset of terrestrialization ~500 million years ago. Then the third phase set in—the end of stasis and the climb [16,17] of O_2_ to todays value of 21 % by volume (100 % PAL).

The origin of O_2_ is largely undisputed. O_2_ is the product of water oxidation in the OEC of photosystem II [18–20]. The mechanism giving rise to high O_2_ at the origin of land plants is also largely undisputed. It is increased carbon burial caused by land plants [16,17]. The question of which mechanism kept O_2_ low during the 2 billion years following the GOE is, by contrast, heavily disputed. Geochemists have presented a number of different models involving geological or geochemical processes to account for the course of Earth oxygenation [21–23]. Here we suggest that the accumulation of O_2_ in Earth history occurred by purely biological, not geochemical mechanisms. Moreover, we propose that the overall contours of O_2_ accumulation in Earth history were governed by only three enzymes. The frequency with which new geochemical mechanisms are suggested to account for the delayed course of O_2_ accumulation indicates that they cannot all be simultaneously correct. Accordingly, it could be worthwhile to at least consider the possibility that alternative, biological mechanisms—vast armies of cells brimming with highly-specific catalysts—were at work instead. And even if one disagrees with our proposal, one can learn something about the enzymes that caused an atmosphere to fill up with O_2_ and the mechanisms that convey protection of essential enzymes against damage caused by O_2_.

Data from [3–12,16,17]. Note that ultra-light carbon in 3.8 Ga rocks is usually interpreted in the context of archaeal methanogens [24,25], but bacterial acetogens carry an indistinguishable isotopic signal [26]. Photosynthesis first evolved to use reductants other than water, such that oxygen was not produced. The origin of oxygenic photosynthesis at the GOE coincides with the evolution of cyanobacteria, which generated the global supply of O_2_ via one single enzyme and one single enzyme activity: the conserved Mn-containing oxygen evolving complex of photosystem II. A broken line following the end of the GOE indicates the Lomagundi excursion [27]. The reasons why O_2_ levels then remained near the Pasteur point for 1.8 billion years are still discussed. In addition to numerous geological causes [21–23], one biological cause [28] for the existence of the boring billion has been proposed—the inhibition of nitrogenase by oxygen. With the advent of land plants, nitrogen fixation and photosynthesis became spatially separated, on one hand, and the activity of cellulose synthase resulted in extensive burial of carbon in a nitrogen-free form, on the other. This allowed for oxygen levels to rise to present levels (100 % PAL) starting about 500 MY ago.

## 2 One enzyme in one lineage produced O_2_ at the GOE

The source of environmental O_2_ on Earth is oxidation of water by the OEC in photosystem II of the photosynthetic electron transport chain of cyanobacteria and their descendants, plastids [5,29]. Photosystem II with its OEC is the only enzyme that results in a net increase of O_2_ in culture media or the environment. Recent proposals challenge this view by suggesting that non-enzymatic reactions of sand and water produced geochemically derived H_2_O_2_ as a putative source of abiotic O_2_ [30–32]. Those proposals face a near insurmountable hurdle, however. In the presence of dissolved Fe (II), the half-life of H_2_O_2_ is only 0.7 s [33] because of the Fenton reaction, which converts the extremely reactive oxidant H_2_O_2_ into the even more reactive hydroxyl and hydroxyperoxyl radicals. These react with almost anything organic in the environment. Earth’s earliest aqueous environments were replete with Fe (II) [34]. As a result, H_2_O_2_ cannot have accumulated to levels that would support O_2_ production to a degree that would impact the environment or ecosystems [33,35]. Cells can admittedly be forced to grow on H_2_O_2_ [36], but there are no known ecosystems that live from geochemical H_2_O_2_.

Photosystem II of oxygenic photosynthesis is a light-driven water-–plastoquinone oxidoreductase that is conserved in cyanobacteria, eukaryotic algae, and plants. As shown in Fig. 2, the cyanobacterial enzyme is a dimer, which contains the photochemical reaction center housed in a core of two five-helix transmembrane proteins (D1 and D2) that ligate chlorophyll and phaeophytin, spanning the photosynthetic membrane. The two core subunits are surrounded by two additional large subunits (CP47 and CP43) composed of six membrane-spanning helices each. They bind several chlorophyll molecules which funnel the absorbed light energy towards the reaction center. Light-driven electron transfer from the excited reaction center P680* proceeds through pheophytin, until eventually two plastoquinone molecules bound close to the inner, aqueous, cytoplasmic/stromal N-phase – the acceptor side, are reduced [20]. The plastoquinone Q_A_ is a fixed one-electron carrier. It transfers two single electrons to the Q_B_ plastoquinone, which diffuses into the membrane in its reduced quinol form to become part of the PQ pool. In order to reestablish its neutral state, the photooxidized chlorophyll at the reaction center acquires electrons that are released from water within the OEC at the donor (lumen) side, adjacent to the membrane surface that faces outward to the aqueous P-phase.

The OEC is the catalyst that extracts molecular oxygen from water, releasing it into the atmosphere. In all cyanobacteria and plastids the OEC is an inorganic cluster, Mn_4_CaO_5_, bound to side chains of photosystem II polypeptides [19]. Acquisition of this metal cluster allowed photosynthesis access to an unlimited supply of a novel electron donor – water. This innovation changed the world.

Where did the OEC come from? There are minerals with precisely its atomic stoichiometry [37]. It seems likely that one such mineral – birnessite – accumulated as a result of its deposition by early oxygenic phototrophs [38]. Photosystem II will accept electrons from dissolved Mn^2+^ [38], thereby generating higher oxidation states of manganese. The sequential oxidation of the four Mn atoms in the OEC via a tyrosine radical, itself produced by photooxidized chlorophyll, accounts for the four-fold periodicity in the flash yield of oxygen evolution [13] reported by Joliot [39]. The mechanism of formation of the O–O bond involves transient distortion of the cubane structure of the OEC, bringing a water molecule or hydroxide ion into close proximity with one of its μ-oxo linkages [14,40,41]. Chernev et al. [38] propose that Mn^2+^ in solution acted as an early electron donor to a type II photosynthetic reaction center, and that the resulting protein-bound manganese oxide nanoparticles were precursors of the OEC. This view is consistent with geochemical evidence of manganese-rich sedimentary layers that are dated at 2.5 Gy—at, or immediately before, the Great Oxidation Event (GOE) [42].

## 3 Geochemical mechanisms proposed for the persistence of low O_2_ following the GOE

Since the realization that oxygen levels were low throughout the Proterozoic, a recurring question about O_2_ in evolution has been: Why did O_2_ levels remain low for 2 billion years following the GOE? There have been a number of proposals to account for low Proterozoic O_2_ involving geochemical mechanisms. One proposal posits a steady supply of geochemical reductants from within the Earth, such as Fe^2+^ or S^2-^, that consumed O_2_ either enzymatically or non-enzymatically. The reductants were emitted from the mantle at rates and in amounts that aligned rather precisely with cyanobacterial O_2_ production so as to keep O_2_ levels low and constant for 2 billion years without variance or interruption [43,44].

A second proposal has it that anoxygenic phototrophs out-competed oxygenic cyanobacteria for light or for nutrients such as phosphorus [45]. Yet in order to outcompete cyanobacteria for any nutrient, the ‘more successful’ anoxygenic phototroph would first require more carbon for cell mass than cyanobacteria, meaning a supply of reductant for CO_2_ fixation that is more abundant than water. There is no environmental reductant more abundant than water.

A third proposal posits that nutrient limitations, in particular molybdenum (Mo), led to limited O_2_ production by limiting photosynthetic biomass [46–48] via limited biogenesis of nitrogenase. Yet when molybdenum is limiting, cyanobacteria can use either Fe or V as a substitute for Mo in their alternative nitrogenases and immediately resume growth unimpaired [49], such that microbes readily overcome the theory of Mo limitation by biochemical routes. In that context, marine iron was not limiting for any prolonged phase of the Proterozoic [35].

A fourth proposal has it that animals affected the degree of mixing between nutrient-rich reservoirs and the photic zone, for example, through animal burrowing activity [50,51] or by grazing activity of early animals to improve light penetration into the photic zone, increasing O_2_ production to end the Pasteurian epoch [52]. This would address why oxygen levels rose at the end of the Proterozoic, but not why O_2_ stayed low during the boring billion.

A fifth proposal has it that no mechanism at all is required to account for Earth’s stepwise oxygenation, it is an inherent property of biogeochemical cycling as calculated by a mathematical model [53]. This would not explain why O_2_ levels started to rise again near the Ediacaran phase of the Neoproterozoic.

A sixth proposal invokes changes in the magnitude of tides, which resulted in the deceleration of Earth’s rotation and subsequent changes in day length that would impact O_2_ accumulation once it set in [54], thereby limiting the planet’s oxygenation.

The foregoing geochemical proposals fail to address the issue of how these environmental mechanisms, individually or in concert, could operate to produce a *constant and low* O_2_ level across 2 billion years of cyanobacterial existence. This is particularly problematic from the biological standpoint because bacterial growth is exponential. A mere 144 doubling times would give rise to a bacterial culture that outweighs the Earth unless something limits the growth process. The biologist looks for mechanisms that limit cyanobacterial growth as opposed to geochemical mechanisms that consume oxygen or that alter the Earth’s rotation about its axis. In addition, any geochemical mechanism would need to account for the observation that O_2_ started to rise to modern levels around the time that animals and land plants appeared. The geochemical mechanisms differ sharply in their functional details but they have one element in common: they accept the evidence that one, single cyanobacterial enzyme generated O_2_ at the GOE, the OEC of photosystem II. Given a biological mechanism as the source of O_2_, biological mechanisms to limit it have not been sufficiently explored.

## 4 Nitrogenase is inhibited by O_2_ through damage to FeS clusters

If *one* cyanobacterial enzyme could make all of Earth’s O_2_, is it also possible that *one* cyanobacterial enzyme could also limit Earth’s O_2_ levels for 2 billion years? Yes, and the enzyme is nitrogenase [28]. Nitrogenase is an enzyme that catalyzes reduction of nitrogen (N_2_) to ammonia (NH_3_). Nitrogenase is inhibited by O_2_. The mechanism of inhibition is O_2_-dependent dissociation of FeS clusters. There are three different FeS clusters in nitrogenase [55,56] (Fig. 3). All three are essential for the protein’s activity because they are involved in the protein’s conduction of 8 single electrons from ferredoxin to N_2_, and all three FeS clusters are rapidly oxidized by O_2_. The reason that FeS clusters in proteins, if they are solvent-exposed, are so sensitive to the oxygen diradical is that oxygen is a strong oxidant that readily accepts single electrons. FeS clusters in active electron transport chains always transfer single electrons, hence they readily convert the O_2_ diradical to the extremely reactive superoxide radical ${\text{O}}_{2}^{•-}$, which can cause irreparable damage to the cluster, forcing resynthesis of the enzyme. Oxygen-derived reactive species such as superoxide and hydrogen peroxide can also form by the interaction of reduced flavins with oxygen, and they act in a similar fashion as O_2_, oxidizing solvent-exposed low-potential FeS clusters. If high O_2_ is persistent, the newly synthesized nitrogenase enzyme is inactivated by the same recurrent mechanism.

For example, the nitrogenase from *Azotobacter chroococcum* and other sources is rapidly inactivated by O_2_ [57,58]. The very O_2_-sensitive 4Fe4S cluster of the Fe protein (FeP) is afforded some degree of protection from O_2_ oxidation by binding of the Shethna protein, which has a 2Fe2S cluster that covers the 4Fe4S cluster of FeP [56,57] under oxidizing conditions. But that protection is ephemeral. In the presence of air, the 4Fe4S cluster in the Fe protein (FeP, NifH) of *Azotobacter* nitrogenase has a half-life of about 30–60 s while the FeS clusters in the catalytically active MoFe protein (NifDK) have a half-life of about 5–10 min [58]. Both components of nitrogenase precipitate immediately if the FeS clusters are degraded by O_2_ (M. Ribbe, pers. comm.), indicating that the protein undergoes irreversible conformational changes as a result of FeS cluster loss.

## 5 Nitrogenase in cyanobacterial cultures limits O_2_ accumulation to no more than 10 % PAL

Nitrogenase inhibition by O_2_ stably limits O_2_ accumulation in the gas phase above cyanobacterial cultures [61–68]. As an example, nitrogenase from *Plectonema* is inhibited by 41 % at 1 % [*v*/v] O_2_, by 71 % at 2 % [v/v] O_2_, and is completely inhibited by 10 % [*v*/v] O_2_; the inhibition is irreversible, and below 1 % [*v*/v] O_2_ nitrogenase remains measurably active [61]. This inhibition autoregulates O_2_ levels in the air above a cyanobacterial culture, as long as it *lacks* mechanisms to protect nitrogenase from O_2_, as seen with nitrogen-fixing cyanobacteria such as *Plectonema*. When *Plectonema* cultures are grown under a CO_2_-N_2_ atmosphere with N_2_ as the sole N source, with sufficient CO_2_ and light, they grow and accumulate about 0.5 to 1 % [*v*/v] O_2_ but no more than 2 % [v/v] O_2_ (10% PAL) in the gas phase above the culture [61–68]. This is because air with 1 % O_2_ inhibits nitrogenase activity by about 41 % while air with 10 % O_2_ inhibits nitrogenase activity completely [61]. This autoregulatory circuit (Fig. 4) maintains a low O_2_ level above the cyanobacterial laboratory culture to a maximum of 2 % [v/v] (or 10 % PAL). This O_2_ level remains constant during prolonged cultivation. If nitrogenase is inactivated by higher O_2_ partial pressure, there is no fixed N to support cell mass accumulation hence O_2_ production, which has to balance CO_2_ and N_2_ fixation (cyanobacteria are 50 % C and 10 % N by weight whereby CO_2_ consumes four electrons to become carbohydrate and N_2_ consumes eight electrons to become ammonia via nitrogenase). With less O_2_, nitrogenase activity increases, allowing more CO_2_ fixation hence more O_2_ production. The resulting circuit maintains O_2_ in the atmosphere above the culture below 2 % O_2_ or 10 % PAL and is governed by O_2_-sensitive FeS clusters in nitrogenase. This feedback loop is outlined in Fig. 4.

The reason that nitrogenase inhibition limits O_2_ accumulation above cyanobacterial cultures is because the enzyme that provides N for growth (nitrogenase) is produced by the same organism that produces O_2_ at the OEC, and fixed N is produced in amounts that scale linearly with the OEC and with the O_2_-producing electron transport chain. Fixed N is required to make the proteins and pigments of the photosynthetic apparatus. The factor that limits O_2_ production (nitrogenase) is always present in a strict stoichiometry with the OEC. Its inhibitory effect therefore scales globally. Nitrogenase inhibition by O_2_ operates wherever O_2_ is encountered in the environment. Nitrogenase regulates O_2_ levels above a cyanobacterial culture in water. It presents one single point of attack by O_2_ that limits O_2_ production. Other factors could enter into the feedback mechanism (Fig. 4) that reduce O_2_ levels further, but no mechanism other than protection of nitrogenase from O_2_ (see next section) could allow Proterozoic O_2_, produced in the ocean for lack of life on land, to accumulate to levels higher than 10 % PAL. One might interject that cyanobacteria are not the only organisms in the Proterozoic, and many other prokaryotes fix N_2_ [69]. But all known nitrogenases are inhibited by O_2_, and there is no enzymatic alternative to nitrogenase for N_2_ fixation, such that O_2_ inhibition of nitrogenase operates at a global scale wherever cells are in contact with the atmosphere (and hence the photic zone). O_2_ inhibits all nitrogenases, but the inhibition of cyanobacterial nitrogenase is what kept O_2_ below 10 % PAL for 2 billion years.

## 6 Nitrogenase inhibition limited atmospheric O_2_ accumulation to a maximum of 10 % PAL

As it relates to Earth’s Proterozoic atmosphere, there is no reason why nitrogenase inhibition would not operate on a global scale to limit O_2_ levels in Earth’s atmosphere [28]. The mechanism—nitrogenase inhibition by O_2_—operates independently of environmental factors, as long as light, CO_2_, water and N_2_ are available (in addition to trace ions including Mo, V, or Fe in Proterozoic oceans), and this inhibition can be observed directly in laboratory cultures. Nonetheless, the mechanism that leads to stable O_2_ in the gas phase above cultures is observed directly only in those cyanobacteria that possess no mechanisms of protecting nitrogenase from O_2_. Hence one might interject that the nitrogenase-inhibition mechanism of keeping Proterozoic oxygen low (Fig. 4) runs counter to the observation that many cyanobacteria have evolved mechanisms to protect nitrogenase from O_2_, and that such mechanisms, had they evolved in the Proterozoic, would have allowed O_2_ to accumulate to modern levels despite the inhibitory effect of O_2_ on nitrogenase. That argument fails, however. To understand why, one must consider the biological mechanisms that protect nitrogenase against O_2_ in cyanobacteria, and how they work.

**Fig. 1:**
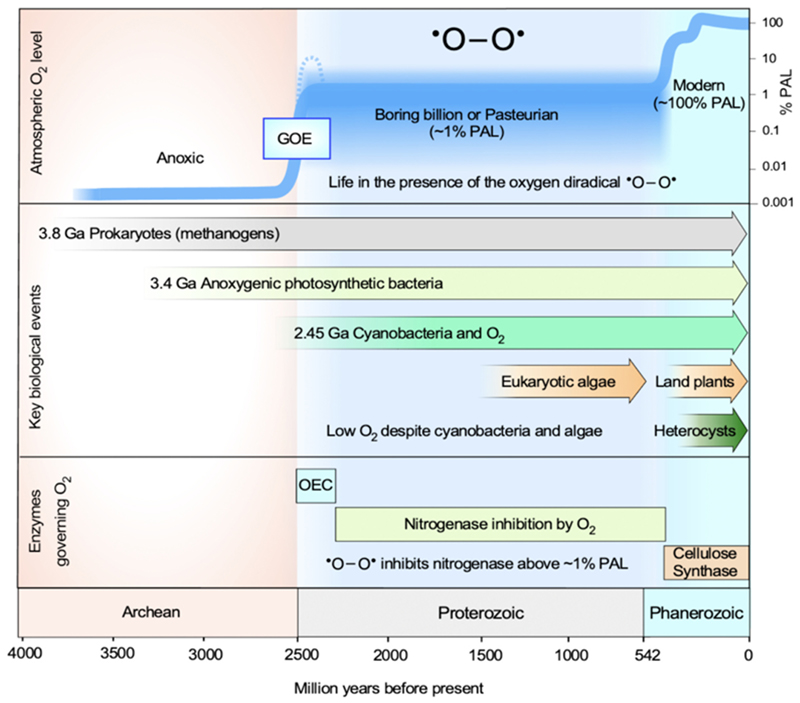
A timeline of Earth history with the rise of O_2_ governed by three enzymes and the appearance of some relevant groups of eukaryotes and prokaryotes.

**Fig. 2:**
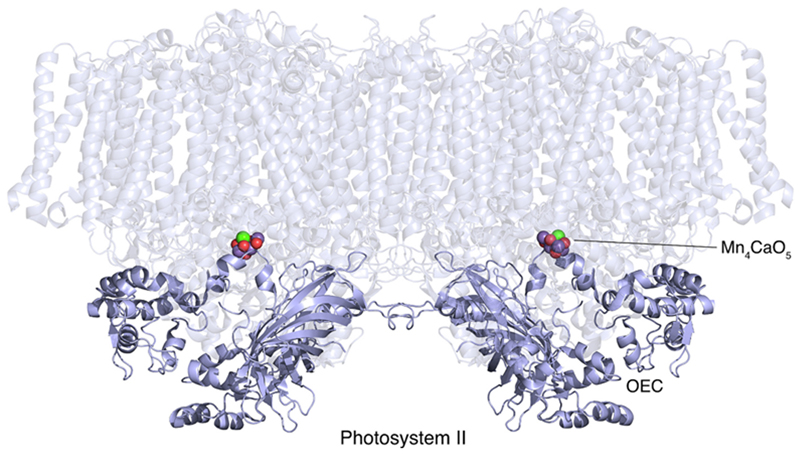
The OEC in photosystem II. The image is of cyanobacterial photosystem II (PDB ID: 7D1T) [18]. The transmembrane subunits are shown with high transparency, while the membrane peripheral, extrinsic proteins PsbO (manganese stabilizing protein), PsbU and PsbV (cytochrome *c*_550_) are highlighted in opaque purple [19,20]. The Mn_4_CaO_5_ cluster constitutes the OEC, the active site of water oxidation and oxygen evolution. Atoms in the Mn_4_CaO_5_ cluster drawn as spheres: red, O; purple, Mn; green, Ca. The figure was created using The PyMOL Molecular Graphics System, version 2.5.4, Schrödinger, LLC.

**Fig. 3:**
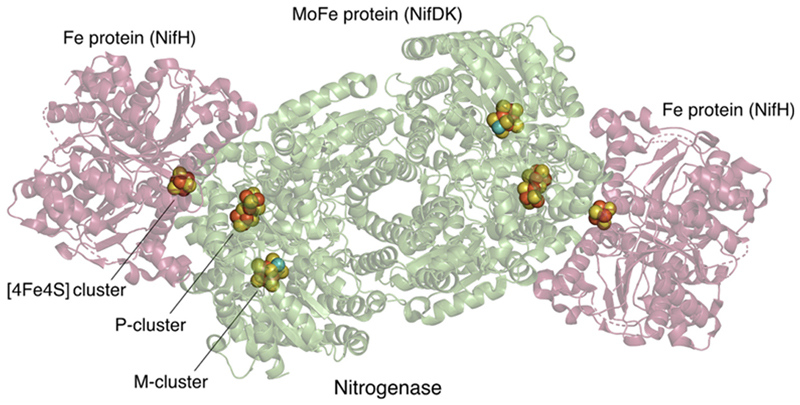
Structure of nitrogenase (PDB ID: 1G20) [59] highlighting FeS clusters. The nitrogenase MoFe protein (NifDK), an α_2_β_2_ tetramer, is shown in green, while the two Fe proteins (NifH), each a γ_2_ dimer, are in red. The metal clusters are shown, namely the M-cluster [MoFe_7_S_9_] and the P-cluster [Fe_8_S_7_] of the MoFe protein, as well as the [Fe_4_S_4_] cluster of the Fe protein [60]. Atoms as spheres: red, Fe; yellow, S; teal, Mo. The figure was created using The PyMOL Molecular Graphics System, version 2.5.4, Schrödinger, LLC.

**Fig. 4:**
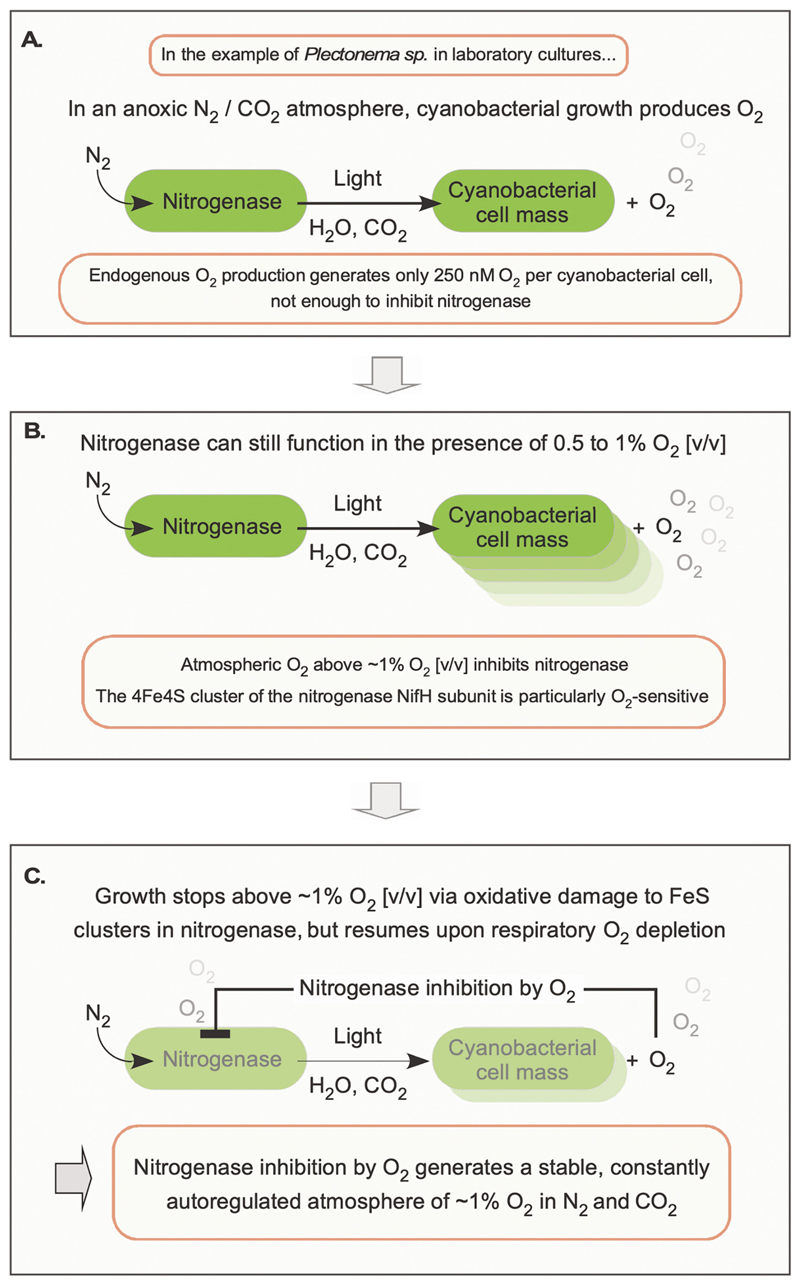
Nitrogenase inhibition by O_2_ limits atmospheric O_2_ to the Pasteur point during the boring billion (or Pasteurian). See text. Modified from Allen et al. [28].

An important observation is that the geological record itself indicates that cyanobacteria evolved nitrogenase O_2_ protection mechanisms late in evolution, after the origin of land plants and at a time when O_2_ was already rising to approximately modern levels. This is because the oldest fossil heterocysts (specialized cells that protect nitrogenase from O_2_) are from the Rhynie Chert and only 418 million years old (shown in Fig. 1) –– younger than the oldest land plants [70]. Cyanobacteria have a long and comparatively good fossil record [71]. There are reports for cyanobacteria lineages that produce akinetes (resting spores) in sediments much older than the Rhynie Chert [72,73]. Because akinetes are made by the same cyanobacterial lineages that make heterocysts, there is an indirect argument to be made that the presence of akinetes serves as a proxy for the presence of heterocysts. But heterocysts themselves appear much later in the fossil record, together with land plants in the Rhynie Chert [70]. The recent origin of heterocysts, 2 billion years after the origin of O_2_ production, and coming in the wake of rising O_2_ (Fig. 1) after the origin of land plants, clearly suggests that cyanobacteria evolved mechanisms of nitrogenase protection against O_2_ in response to environmental (atmospheric) O_2_ levels that were persistently above the 1 % O_2_ threshold for nitrogenase inhibition [28] around 500 MY ago, rather than for protection against endogenous O_2_ production, which has been in effect for 2.5 billion years. Do other O_2_ protection mechanisms lead to the same conclusion? Yes.

Intracellular O_2_ concentrations generated by endogenous O_2_ production by cyanobacteria have been measured for many different lineages. In this context, endogenous means “O_2_ produced within the cell” whereas environmental refers to ambient O_2_ that comes from outside the cell. Endogenous O_2_ production generates intracellular O_2_ concentrations of only 25 nM in *Gloeobacter*, which lacks thylakoids, up to 250 nM in filamentous forms [74], 100-fold lower than levels of O_2_ that inhibit nitrogenase. That is important because in the context of low Proterozoic O_2_, it clearly indicates that cyanobacteria evolved their three developmental mechanisms to protect nitrogenase from atmospheric (environmental, ambient) O_2_ rather than to protect nitrogenase from endogenous O_2_ production.

## 7 How do cyanobacterial nitrogenase protection mechanisms against O_2_ operate?

To appreciate the importance of low endogenous O_2_ production by cyanobacteria, we need to look at how the mechanisms that protect nitrogenase from O_2_ actually work. In a nutshell, they do not work by shutting down endogenous O_2_ production (which is not sufficient to inhibit nitrogenase anyway, as we just saw), rather they generate protection against environmental O_2_ that enters the cell from outside. There are three main biological strategies of nitrogenase protection known among cyanobacteria: differentiated cells called heterocysts [75], filament bundles as in *Trichodesmium* (also called diazocytes) [76], and light-dark cycles (diel cycles) in unicellular forms. How do these mechanisms protect nitrogenase from O_2_?

Heterocysts are large, thick-walled cells in filamentous cyanobacteria that express nitrogenase and that differentiate during growth in response to N limitation [77,78]. They are the only site of N_2_ fixation for heterocyst-forming cyanobacteria. It has long been known that heterocysts do not express photosystem II [79], but that alone is not the mechanism of protection against O_2_. Rather, it is the thick cell wall and surrounding sheaths consisting of glycolipid layers that generate a barrier to diffusion against ambient (environmental) O_2_ [78], in addition to high affinity respiratory (O_2_-consuming) activities in the heterocyst plasma membrane [80]. Mutants defective in the synthesis of glycolipids of the heterocyst wall form heterocysts that do not protect nitrogenase from O_2_ [77,80]. Heterocysts protect nitrogenase from O_2_ levels exceeding those of the Proterozoic –– they arose in *response* to high O_2_, not as a means to generate high levels of O_2_, the environmental toxin that they handle.

Filament bundles are well studied in *Trichodesmium* [76]. Earlier views held that *Trichodesmium* bundles allowed central cells within the bundles to fix N_2_, a spatial protection from O_2_ [76]. Newer views have it that the protection is temporal in nature [76], such that many or all cells in bundles can fix N_2_ with O_2_ protection involving avid O_2_ consumption in a temporally regulated photosynthesis-N_2_ fixation cycle [81]. On a day-to-day level, *Trichodesmium* cells perform photosynthesis and accumulate carbohydrate reserves in the early daylight hours. At midday, photosynthetic activity is decreased and N_2_ fixation sets in [82]. The energy for the energy intensive N_2_ fixation process (16 ATP per N_2_) comes from vigorous O_2_ respiration of accumulated photosynthate at the plasma membrane [83,84]. This temporal separation of photosynthesis provides a low-oxygen environment in the cytosol, because O_2_ is consumed at the plasma membrane before it diffuses into the cell interior.

Diel protection of nitrogenase against O_2_ is well-studied in unicellular N_2_ fixing cyanobacteria with a light-dark N_2_ fixation cycle such as diazotrophic *Cyanothece* [85,86] or *Synechococcus* strains [87]. In diel N_2_ fixation, photosynthesis takes place in the light, nitrogen fixation takes place in the dark, however, the lack of endogenous O_2_ production in the dark is immaterial because endogenous O_2_ production is, on its own, 100-fold below that required to inhibit nitrogenase. Environmental O_2_ is the threat to nitrogenase. The mechanism of O_2_ protection is similar to that in *Trichodesmium* in that photosynthate is accumulated in the light and nitrogenase activity is expressed when photosynthesis is repressed, but in the dark (diel) rather than in the light (filament bundles). In the diel protection mechanism, nitrogen fixation is, as in *Trichodesmium*, supported by vigorous respiration of exogenous O_2_ at the plasma membrane in order to supply the N_2_ fixation process with ATP and electrons. Vigorous respiration at the plasma membrane in the dark leaves the cytosol O_2_-depleted, and N_2_ fixation proceeds.

Depletion of cytosolic O_2_ by respiration at the plasma membrane is central to several mechanisms that protect nitrogenase from O_2_. It is a well-known mechanism that is employed by many N_2_ fixing bacteria other than cyanobacteria, such as soil dwelling N_2_ fixers and root nodule-forming bacteria of legumes (reviewed by Gallon [88]). These bacteria also perform avid respiration at the plasma membrane during N_2_ fixation, but without the diel cycle. This is the case for *Azotobacter* for example, whose avid O_2_ consumption keeps O_2_ levels in the cytosol low enough for efficient N_2_ fixation in the presence of ambient environmental O_2_, expressing a high affinity terminal oxidase with a nanomolar substrate affinity for O_2_ [89] and NADH oxidases, which consume O_2_ rapidly enough to allow high nitrogenase activity even in the presence of high exogenous O_2_ [88].

## 8 How did nitrogenase protection mechanisms impact proterozoic O_2_ levels?

One might argue that cyanobacteria could have possessed nitrogenase protection mechanisms in the Proterozoic, allowing O_2_ to rise or fall independently of nitrogenase inhibition, or that cyanobacteria could have just invented nitrogenase protection mechanisms that would have allowed them to produce much higher levels of O_2_. Such arguments present, however, a non sequitur because the mechanisms of nitrogenase protection against O_2_ that arose in cyanobacteria (and soil bacteria) arose to protect against O_2_ levels that were not present in the Proterozoic. Furthermore those mechanisms would have generated O_2_ levels that were not present in the Proterozoic.

Nitrogenase-dependent O_2_ limitation puts an upper bound of ~10 % PAL on Proterozoic oxygen levels, consistent with current observations from geochemical record (Fig. 1). But it places no lower bound on atmospheric O_2_ levels during the Proterozoic, readily accommodating periods of local or global anoxia, which would by themselves not abolish primary production, as long as sunlight reached the oceans. Could nitrogenase-dependent O_2_ limitation accommodate Proterozoic O_2_ oases, as has been proposed [90]? Yes: in an environment that harbors a rich reservoir of fixed N (organic N for example) that decouples N supply from nitrogenase, local oxygen oases would be possible, but only until the N supply became exhausted, after which nitrogenase inhibition would resume.

In addition, nitrogenase inhibition would directly explain why the appearance of eukaryotic algae, today the most prolific primary producers in the oceans [91], did not lead to an increase in atmospheric O_2_. No eukaryotes fix N_2_, they are all dependent upon N provided by nitrogenase, and this is one reason why the plastid ancestor could have been a nitrogen fixing cyanobacterium [92]. Proponents of geochemical mechanisms to explain low O_2_ would have to advance corollaries in order to account for the missing contribution of eukaryotes to Proterozoic O_2_. By contrast, O_2_-dependent nitrogenase inhibition predicates that photosynthetic eukaryotes, whose fossil record appears to extend as far back as 1.6 Ga [93], could not have pushed Proterozoic O_2_ levels beyond 10 % PAL in any case, because nitrogenase was the sole source of nitrogen, whether environmentally allocated to prokaryotes, to eukaryotes, or both. In addition to accounting for low Proterozoic O_2_ per se, N allocation could thus also help to explain why eukaryotic algae existed for almost a billion years without rising to dominance [8]: they had to live on nitrogen that prokaryotes left over.

## 9 Cellulose synthase caused the late rise of O_2_ to current levels

**Fig. 5:**
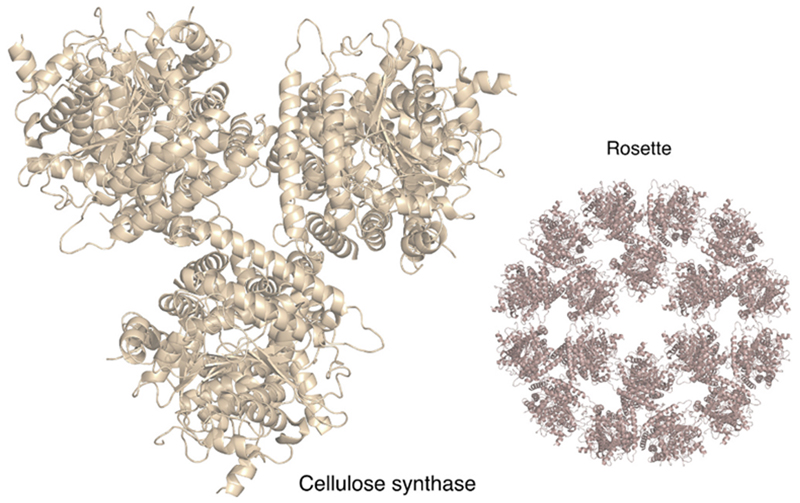
Cellulose synthase. The cellulose synthase homotrimer is shown (PDB ID: 6WLB) [97], whereby several of these trimers further organize in the membrane into a rosette – a hexamer of trimers [97]. The structures are radially symmetrical. With the evolution of land plants and subsequent large-scale carbon burial due to cellulose synthesis and the physical separation of N_2_ fixation in the ground from CO_2_ fixation (and O_2_ production) in leaves, oxygen levels rose towards modern levels (see text). The figure was created using The PyMOL Molecular Graphics System, version 2.5.4, Schrödinger, LLC.

Nitrogenase inhibition could have kept atmospheric O_2_ levels globally low and constant for 2 billion years. How did O_2_ levels overcome nitrogenase inhibition? By the activity of an enzyme, cellulose synthase (Fig. 5), as life was starting to emerge on land about 500 million years ago. The exact timing of the final surge in O_2_ evolution is still discussed, but newer reports [17] suggest that deep-ocean oxygenation occurred only 541 million years ago and perhaps less than 420 million years ago. This conclusion is in line with other recent studies [16] that implicate land plants as the cause of late O_2_ rise in Earth history (for a recent review see [7]). Current estimates have it that the first land plants arose roughly 500 MY ago [94], which is compatible with evidence from fossil spores indicating the presence of early land plants [95,96].

As oxygen started to rise to modern levels roughly 500 MY ago, nitrogenase inhibition by O_2_ no longer limited atmospheric O_2_ because land plants (which generate about half of all O_2_ today) perform photo-synthesis in aerial organs that are physically removed from their source of nitrogen: N_2_ fixing microbes in the ground. Land plants amplified one simple enzyme activity that is also present in prokaryotes [98], cellulose synthase, and optimized it in terms of fiber production in order to generate the main component of land plant cell walls: cellulose. Cellulose is polymeric glucose, C_6_H_12_O_6_, it is also produced in the cell walls of roots, but it is mainly produced from UDP-glucose in the cell walls of stems and leaves, the tissues in which land plant photosynthesis takes place.

The OEC in the leaves of land plants generates O_2_ in the process of supplying the electrons from H_2_O to the photosynthetic electron transport chain and then to CO_2_ fixation in the Calvin cycle, which generates phosphorylated sugars. These then form activated glucose monomers that are polymerized upon exiting the cell as cellulose (and other polymers, but mainly cellulose). Land plants have access to more light than their water-column-dwelling algal ancestors did. While algae can redirect electrons to hydrogenases as a safety valve for excess electron flow under anoxic conditions, land plants lost that ability because they converted their FeS cluster-containing hydrogenases into FeS cluster-containing oxygen sensors that operate much in the same way as aconitases do [99] or the *E. coli* redox sensitive transcriptional regulator FNR does [100], namely via O_2_-dependent or ${\text{O}}_{2}^{•-}$-dependent removal of Fe from FeS clusters [101].

## 10 Oxygen uptake in photosynthesis

Because land plants lack safety valves that export excess electrons stemming from the photosynthetic electron transfer chain, they direct electrons to CO_2_ and deposit the product outside the cell, typically as cellulose. However, some electrons in land plants do not end up as cell mass. They make transient, reactive oxygen species, and are eventually returned to H_2_O. During photosynthesis, plant-type ferredoxins transfer electrons from the acceptor side of chloroplast photosystem I to ferredoxin-NADP^+^ reductase, which then reduces NADP^+^ to NADPH. These plant-type, 2Fe2S ferredoxins can also transfer electrons non-enzymatically to O_2_ to form hydrogen peroxide, H_2_O_2_ [102]. This reaction appears to be unavoidable in vivo, and proceeds by a two-step mechanism in which ferredoxin simultaneously reduces O_2_ to superoxide, O_2_•^–^, and superoxide to hydrogen peroxide, H_2_O_2_ [102]. In the light, isolated chloroplast thylakoids oxidize water to O_2_ that, like NADP^+^, can serve as an electron acceptor for reduced ferredoxin generated by photosystem I [103] and the relative proportion of electrons from Fd that generate H_2_O_2_ vs. NADPH can be determined for different ferredoxins. It has been shown that plant-type, 2Fe2S ferredoxins from spinach, maize, alfalfa and parsley, predominantly support NADP^+^ reduction, while the 4Fe4S ferredoxins from the anaerobes *Chromatium, Chlorobium* and *Clostridium* undergo non-enzymatic reaction with O_2_ more readily than enzymatic NADP^+^ reduction [104]. Ferredoxins are single-electron acceptors and can have differing midpoint potentials depending upon the specific configuration and electronic environment of the FeS cluster within the protein [105]. Before the GOE, 4Fe4S ferredoxins will have participated in anoxygenic photosynthesis and other prokaryotic metabolic processes without danger of reacting with O_2_. Following the GOE, cyanobacterial (and plastid) 2Fe2S ferredoxins may have become more specific as electron donors to NADP^+^ in response to selection for avoidance of spontaneous reduction of O_2_. The “plant-type” 2Fe2S ferredoxin may have evolved to be an O_2_-resistant replacement for O_2_-sensitive 4Fe4S ferredoxin [104,106].

In quantitative terms, the non-enzymatic reduction of O_2_ by ferredoxin during photosynthesis is a side reaction, but a significant one in terms of decreasing redox stress for the plant [107–109]. The existence of this ferredoxin shunt to O_2_ as a “pressure valve” for electrons from the photosynthetic electron chain underscores the redox dilemma of the first land plants. Having lost the hydrogenases that their algal predecessors possessed [101], they could no longer shunt “excess” electrons from photosynthesis to protons for H_2_ production. The only viable electron acceptor in leaves is CO_2_. Plants cannot turn off their photosystems. Their only option to maintain redox balance in the O_2_-producing photosynthetic electron transport chain is to reduce CO_2_. Carbohydrates cannot be deposited in unlimited amounts within the cytosol, such that their extracellular deposition is the only option for the main flux of electrons. Cellulose was not only an excellent metabolic end product to maintain redox balance at land plant origin, it supported upright growth in addition, bringing selective benefit to plants that—with the help of cellulose synthase—could overshadow their neighbors in competition for light.

Cellulose synthesis was the main reaction performed by land plants. It altered the global O_2_ budget by allowing plants to synthesize massive amounts of nitrogen-free carbon polymers with the help of catalytic amounts of nitrogen in enzymes. Today land plants comprise over 80 % of the total biomass carbon on Earth [110], almost all of that carbon is cellulose. Once land plants existed, they dominated Earth’s biomass distribution until today [111], and this is predominantly the result of cellulose synthesis.

## 11 Cellulase activity on land is the equivalent to marine primary production

Cellulose can be synthesized in hours. In old trees and wooden artefacts lignified cellulose can remain stable over thousands of years because it presents an insoluble, solid phase substrate that is extremely difficult to metabolize. Cellulose was a keystone component of the terrestrialization process that witnessed the origin of land plants over 450 MY ago. Oxygen started its Cambrian accumulation at a time in which fossil spores indicate the presence of land plants, but the fossilized plants themselves are scarce. In the ocean, primary production does not generate fibers; rather, it mainly generates branched polymers (e.g., alginates) that form gelatinous sheaths. The first land plants were streptophyte algae like *Chara* [112] that have cellulose cell walls. A consequence of cellulose deposition in terrestrial habitats is that cellulose became, by weight, the basis of the terrestrial food chain. As a consequence, enzymes that degrade cellulose—cellulases [113–115]—became the main source of carbon substrates on land. Fiber degradation entails two kinds of processes that are performed exclusively by two groups of microbes. The aerobic process is performed mainly by fungi, in which the end product of degradation is CO_2_ (and fungal cell mass). The anaerobic process is carried out mainly by bacteria and involves the breakdown of the insoluble (solid-phase) cellulose substrate to soluble sugars (mono- and oligo-saccharides) that are fermented to intermediates and fermentation end-products. These compounds serve as the food source for intestinal uptake in animals [116] and, collectively, as the basis for microbial communities in the gut [117], in sediment and in soil, where fiber-degrading bacteria play an essential role [116]. The solubilization of plant fiber on land requires cellulases that can degrade a solid-phase substrate, and their activity returns stably sequestered carbon (cellulose on land) to the ecosystem in the form of biochemically accessible sugars. Cellulase is thus functionally equivalent to primary production in marine environments, because it returns metabolically inaccessible carbon to the food chain.

## 12 Conclusion

Oxygen arose in a world of anaerobes that learned to avoid it, learned to live with it, and/or learned to use it [118]]. The O_2_ produced by cyanobacteria is clear evidence that these bacteria were constantly present in massive amounts in the 2.4 Ga since the GOE. There is no clear evidence that any other group of microbes was present in abundance comparable to that of cyanobacteria [119]. If any group was capable of limiting O_2_ synthesis in terms of mass action, it was cyanobacteria themselves. Consistent with that interpretation, cyanobacteria are the only microbes that can both synthesize O_2_ in significant amounts and concomitantly limit O_2_ accumulation—through nitrogenase inhibition by O_2_. The late rise in O_2_ following the ‘boring billion’ (the Pasteurian epoch) corresponds to terrestrial cellulose deposition made possible by the physical separation of N_2_ fixation (in soil) from CO_2_ fixation in aerial organs of the first land plants. The activities of three enzymes were crucial for O_2_ accumulation during Earth history: the oxygen evolving complex itself; nitrogenase; and cellulose synthase. On land, cellulases mobilize carbon from insoluble cellulose fibers, giving the cellulases of cellulolytic bacteria and fungi an ecological role similar to CO_2_ fixation pathways of marine primary producers. Three enzymes governed the fate of O_2_ in evolution, they changed the world and made it the way it is today.

## Data Availability

No data was used for the research described in the article.
